# Processing and Characterization of Refractory Quaternary and Quinary High-Entropy Carbide Composite

**DOI:** 10.3390/e21050474

**Published:** 2019-05-06

**Authors:** Hanzhu Zhang, Farid Akhtar

**Affiliations:** Division of Materials Science, Luleå University of Technology, 971 87 Luleå, Sweden

**Keywords:** high-entropy ceramic, solid-state diffusion, microstructure, phase evolution, hardness

## Abstract

Quaternary high-entropy ceramic (HEC) composite was synthesized from HfC, Mo_2_C, TaC, and TiC in pulsed current processing. A high-entropy solid solution that contained all principal elements along with a minor amount of a Ta-rich phase was observed in the microstructure. The high entropy phase and Ta-rich phase displayed a face-centered cubic (FCC) crystal structure with similar lattice parameters, suggesting that TaC acted as a solvent carbide during phase evolution. The addition of B_4_C to the quaternary carbide system induced the formation of two high-entropy solid solutions with different elemental compositions. With the increase in the number of principal elements, on the addition of B_4_C, the crystal structure of the HEC phase transformed from FCC to a hexagonal structure. The study on the effect of starting particle sizes on the phase composition and properties of the HEC composites showed that reducing the size of solute carbide components HfC, Mo_2_C, and TiC could effectively promote the interdiffusion process, resulting in a higher fraction of a hexagonal structured HEC phase in the material. On the other hand, tuning the particle size of solvent carbide, TaC, showed a negligible effect on the composition of the final product. However, reducing the TaC size from −325 mesh down to <1 µm resulted in an improvement of the nanohardness of the HEC composite from 21 GPa to 23 GPa. These findings suggested the possibility of forming a high-entropy ceramic phase despite the vast difference in the precursor crystal structures, provided a clearer understanding of the phase transformation process which could be applied for the designing of HEC materials.

## 1. Introduction

High-entropy ceramics (HECs), as a new class of ceramic materials, are developed from the concept of high-entropy alloys (HEAs). In HEAs, multiple principal elemental metals are incorporated to form a single phase alloy or multiphase composites [1,2]. With a similar design concept, HECs consist of multiple principal ceramic compounds such as metallic oxides, nitrides or carbides. In recent work, it has been found that the high entropy effect applies in the HECs system consisting of multiple components [3,4]. Solid solution phases that lack long-range order can be formed attributed to the minimization of Gibbs energy. Christina M. Rost et al. [5] fabricated an entropy stabilized oxide that showed a single-phase face-centered cubic (FCC) structure. Single phase solid solutions have been reported in systems like high entropy borides [6] and high entropy carbides [7,8]. Compared with conventional ceramic materials, single phase HECs show superior mechanical properties contributed by the strain strengthening effect from lattice distortion. For example, the high-entropy carbide fabricated by E. Castle et al. [7] exhibited a single phase FCC structure and enhanced hardness (36.1 GPa) compared to all the component carbides. 

Due to the high melting point of ceramic materials, the solid-state pulsed current processing (PCP) is the economically preferable processing route as it offers relatively low sintering temperatures and short sintering times [9]. PCP utilizes pulsed current, directional pressure, and high vacuum during sintering and offers the advantage of providing homogenously densified materials [10,11]. Due to the strong covalent bonding and complex crystal structures of refractory ceramics, the synthesis of high entropy ceramics has been performed with ceramic precursors containing one nonmetal element (for example carbide, nitride or boride) and a similar crystal structure [6,7,12,13] using PCP. The selection approach of the ceramic precursors minimizes the geometrical difference among the starting ceramic precursor materials and achieves a single-phase high-entropy phase during PCP [14]. 

Our previous work on the high-entropy ceramic composite shows the possibility of forming a single-phase hexagonal structure from carbide precursors with a vast difference in the crystal structures (FCC, hexagonal and rhombohedral) and two nonmetal atoms (carbon and boron), attributing to the independent diffusion of metal and nonmetal atoms during the PCP processing [14]. However, the phase formation rules in a high-entropy ceramic system are still elusive and how B_4_C influences the diffusion process remains unclear. In order to develop a better understanding of the formation mechanism of the high-entropy ceramic phase, a systematic study was established and conducted in this work. Quaternary HEC composite was processed from four refractory carbides, HfC, Mo_2_C, TaC, and TaC. The phase evolution on B_4_C addition was studied by investigating the microstructure and phase composition of the five-component carbides. Moreover, due to the importance of starting particle size in solid-state diffusion [15], HEC composites were fabricated from precursors with different particle sizes. The effects of tuning the particles size of the solvent and the solute carbides on the microstructure, phase composition, and mechanical properties are discussed.

## 2. Materials and Methods

HfC (<1.25 μm, Sigma Aldrich, Darmstadt, Germany) and (−325 mesh, American Elements, Los Angeles, LA, USA), Mo_2_C (−325 mesh, Alfa Aesar, Haverhill, MA, USA) and (2.6 µm, Nanografi, Ankara, Çankaya), TaC (−325 mesh, Alfa Aesar, Haverhill, MA, USA) and (1µm, US Research Nanomaterials, Houston, TA, USA), TiC (5 µm, H.C. Starck, Munich, Germany) and (2 µm, Alfa Aesar, Haverhill, MA, USA) and B_4_C (1–7 μm, Alfa Aesar, Haverhill, MA, USA) were utilized to synthesize high-entropy ceramic composites. Starting materials with different particle sizes were mixed following the designed recipe, with a molar ratio of 2:1:2:2:2. The powder mixture was homogenized in a ball milling machine for two hours, using 4 mm stainless steel balls as a milling media and a powder to ball mass ratio of 1:5. The homogenized powder mixture was filled into a graphite die with a diameter of 10 mm and prepressed before sintering. The sintering was conducted in SPS-530ET (Dr. Sinter Spark Plasma Sintering System, Fuji electronic industrial Co., Ltd., Tsurugashima, Japan) in a glovebox. The samples were heated to 1800 °C with a heating rate of 100 °C/min and then held at 1800 °C for 5 min under vacuum. A uniaxial pressure of 60 MPa was applied during the process.

Before the microstructure characterization and phase identification, the samples were cold mounted in epoxy and polished following standard metallurgical sample preparation procedures. The microstructure was observed using a Scanning Electron Microscope JSM-IT300 (JEOL, Tokyo, Japan) operating at an acceleration voltage of 15 kV. The elemental composition was analyzed using energy dispersive spectrometer (EDS) mounted on JSM-IT300 that was calibrated with Cobalt. X-ray diffraction (XRD) was conducted using an X-ray diffractometer (Empyrean, PANalytical, Malvern, UK) with Cu-Kα radiation (wavelength 0.154 nm). The scanning was performed from 5 to 120° (2 Theta), with a step size of 0.02°. The XRD data was investigated using the software PANalytical X’Pert HighscorePlus with the PDF-4 database. The nanohardness of sintered HEC composites was determined using an MTS NanoIndenter XP with a Berkovich diamond indenter. The measurements were performed with a maximum load of 100 mN at ambient temperature in air. The force-displacement curves of the indenter were recorded. Due to different phase distribution and grain sizes in the microstructure of the PCP samples, the nanohardness values for sample 4-HEC and 5-HEC were obtained by performing at least 5 measurements at specific regions based on the optical microscope mounted on MTS NanoIndenter XP, while the hardness values for sample HEC(+) and HEC(fine) were obtained by performing matrixes indentations and calculating the average value from indentations that were not located in porosity. 

## 3. Results and Discussions

### 3.1. Effect of B_4_C Addition into (HfMoTaTi)C

**Figure 1:**
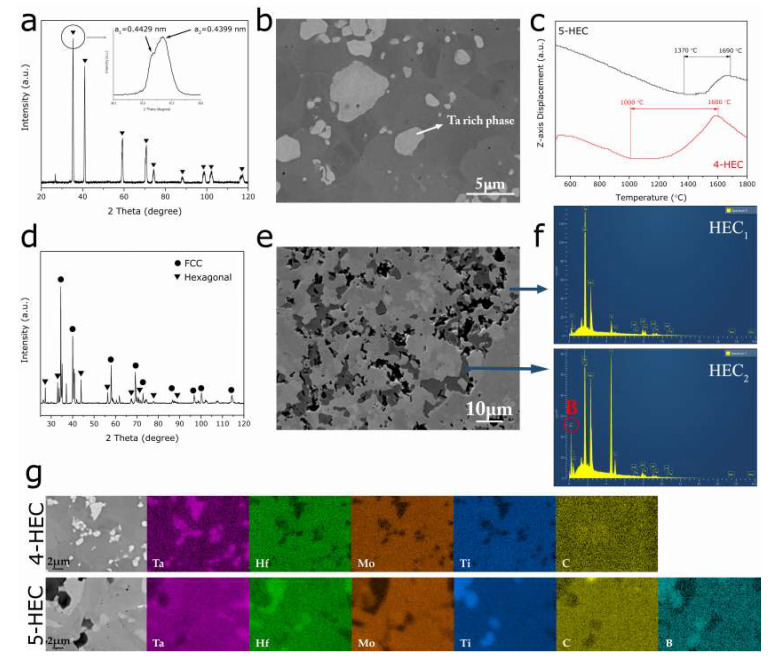
XRD patterns and backscattered electron microstructure of PCP 4-HEC (**a**,**b**) and 5-HEC composite (**d**,**e**), and the EDS mapping analysis (**g**); (**c**) shows the volume change of the material during sintering; (**f**) is the EDS qualitative spectra on different phases of 5-HEC.

**Figure 2:**
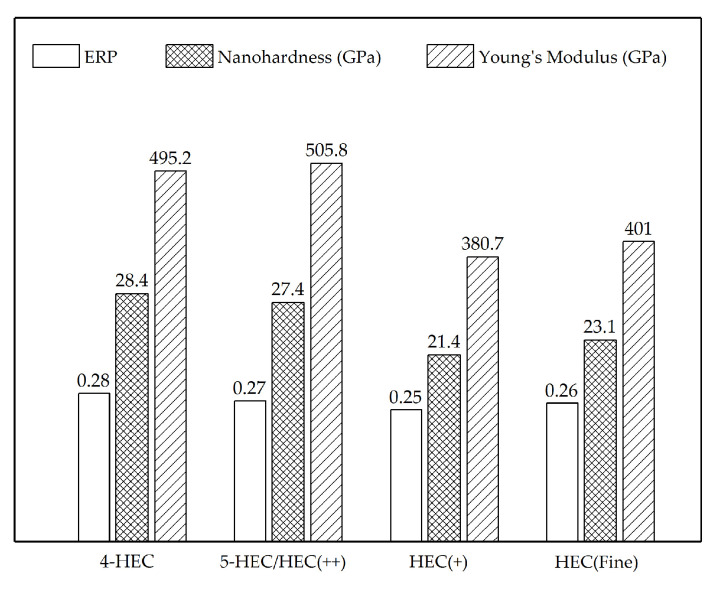
Elastic recovery parameter (ERP), nanohardness and the Young’s modulus of the PCP HECs. The nanohardness values refer to the FCC solid solution in 4-HEC, 5-HEC, and average hardness properties for HEC(+) and HEC(Fine).

The microstructure of the quaternary HEC composite (4-HEC) sintered from HfC, Mo_2_C, TaC, and TiC shows two distinct phases, as shown in Figure 1. The bright phase with a size of 1–5 µm is dispersed uniformly in the dark matrix phase in Figure 1b. Based on the energy-dispersive X-ray spectroscopy (EDS) mapping analysis in Figure 1g, the bright phase is rich in Ta whilst the dark phase contains all constitutional elements Hf, Mo, Ta, Ti and C. Because of the sensitivity of backscattered electron detector (BED) to the atomic number, phases with different densities appear with different contrast in the BED microstructure. The constituent carbides show a vast difference in the densities, from 14.62 g/cm^3^ for TaC, 12.2 g/cm^3^ for HfC, 9.18 g/cm^3^ for Mo_2_C to 4.93 g/cm^3^ for TiC, therefore the consistent contrast of the dark region in the Figure 1b implies that it represents the quaternary ceramic phase containing all constitutional elements Hf, Mo, Ta, Ti, and C. The average atomic ratio of each phase obtained by performing EDS point analysis on several point locations are listed in Table 1. As EDS lacks the accuracy of quantitative analysis of light elements [16], the atomic ratio of the four metals is normalized to Ta. The results show that the bright phase contains Ta and C as the dominating elements and a minor amount of Mo and trace amount of Hf and Ti, while the dark phase contains all four metal elements with a relatively lower amount of Ta. According to the X-ray diffraction (XRD) data in Figure 1a, the PCP 4-HEC composite consists of two face-centered cubic (FCC) crystal structures with similar lattice parameters (a_1_ = 0.4429 nm, a_2_ = 0.4399 nm), as marked in the inset in Figure 1a. The BED microstructure, EDS analysis and XRD data, Figure 1 and Table 1, suggest the formation of high-entropy FCC solid solution containing all constitutional elements. In the previous work on high-entropy ceramic B_4_(HfMo_2_TaTi)C [14], it has been reported that TaC has the lowest metal vacancy formation energy among the precursor carbides, thus it acts as the solvent FCC lattice during the formation of the high-entropy phase [7], i.e., constituent atoms except Ta diffuse into the vacancies in the TaC lattice to form the multicomponent solid solution. Therefore, the Ta-rich phase and the high-entropy phase can be regarded as intermediates in the phase transformation from constitutional carbides to the hexagonal high-entropy ceramic phase. The multicomponent interdiffusion induces TaC lattice distortion, which in this case results in the reduction in the initial lattice parameter of TaC, 0.4460 nm (ICDD reference pattern of TaC: No. 03-065-0282). Based on the quantitative results shown in Table 1, the high-entropy phase with higher content of Hf, Mo, and Ti metal atoms experience intense atomic diffusion compared to the Ta-rich phase. Hence, in the quaternary high-entropy phase, the atomic position exchange between Ta and other metal atoms with similar or smaller atomic radii results in a smaller lattice parameter (0.4399 nm) than the TaC-rich phase (0.4429 nm). Furthermore, the high-entropy phase shows a hardness of 28.4 GPa, which is 23.5% higher than that of the Ta-rich phase (23 GPa) as shown in Figure 2, due to the lattice distortion induced strain strengthening effect. This result is in line with the previous reports on high-entropy materials [6,7].

The curve of *Z*-axis displacement as a function of temperature was recorded during the PCP. As shown in Figure 1c, the decline of *Z*-axis refers to the thermal expansion of the material, while the up-climbing region corresponds to the shrinkage of the bulk volume. The reduction of the volume typically refers to the occurrence of sintering phenomenon where the powder material becomes compacted and forms a densified solid mass. Since the sintering temperature of powder material is normally 2/3–3/4 of the melting point [17], the theoretical sintering temperature for current quaternary refractory carbide mixture should be above 1800 °C. Figure 1c shows that the shrinkage of the four-component carbide system 4-HEC takes place from 1000 °C to 1600 °C, while the same phenomenon for the 5-HEC composite was postponed to a higher temperature range 1370 °C–1690 °C. This indicates that the addition of B_4_C to the carbide system hinders the solid-state atomic diffusion required for sintering in the multicomponent carbides, leading to a delay of the formation of the high-entropy ceramic phase. A detailed investigation on the sintering behavior of ceramic precursors to form high-entropy ceramic composites will be reported later, elsewhere.

The addition of B_4_C to the precursor carbides resulted in the formation of multiple phases during PCP. According to the backscattered electron microstructure in Figure 1e, the PCP 5-HEC composite exhibits three distinct phases. Similar to the 4-HEC, the brightest phase in 5-HEC is rich in Ta, which is coordinated with the fact that Ta has the highest atomic number among the constituent elements, therefore, the Ta-rich phase appears as the brightest phase in the BED microstructure. Two high-entropy solid solutions with different elemental compositions were formed in the five-component system, 5-HEC. The atomic ratio of metal atoms Hf, Mo, Ta and Ti in the gray (HEC_1_) and dark phase (HEC_2_) in Figure 1e are shown in Table 1, with HEC_2_ showing higher content of the solute metal atoms (Hf, Mo, and Ti) in the structure. The higher content of Ti in HEC_2_ agrees more with the darker contrast of HEC_2_ than HEC_1_ in the microstructure, as Ti has the smallest atomic number among the constitutional metal elements. According to the bond dislocation enthalpy (BDE) of transition metal carbides at 298 K [18], Ti-C has the lowest BDE of 423 ± 30 KJ/mol among the solute carbides, while Mo-C_2_ and Hf-C have a BDE of 500 and 540 ± 25 KJ/mol, respectively, and the covalent atomic radii vary as Ti < Mo < Hf. These factors might contribute to preferable diffusion of Ti over Mo and Hf during the formation of the multicomponent solid solution_,_ resulting in the metal content ratio in HEC_2_ phase as Ti > Mo > Hf in Table 1. A pronounced boron diffraction peak in the EDS pattern was revealed at the HEC_2_ phase (Figure 1f), suggesting that HEC_2_ experienced a more intensive diffusion of B atoms than HEC_1_. Additionally, the diffractions peaks of B_4_C were not detected in the XRD diffractogram of the 5-HEC composite in Figure 1d, suggesting the participation of B_4_C in the formation of high-entropy solid solutions. However, the diffusion priority of Ti, Mo and Hf was not observed in the high-entropy phase in the 4-HEC composite and HEC_1_ phase in 5-HEC, suggesting that the incorporation of B_4_C to the transition metal carbides might have promoted the diffusion process of the metal atoms towards a more energetically favorable state.

The XRD data in Figure 1d shows that the 5-HEC composite contains both FCC and hexagonal structured phases. Similar with the 4-HEC, the FCC pattern is generated from diffraction of two FCC crystal structures with similar lattice parameters, including the presence of an FCC Ta-rich phase. Based on the aforementioned discussions, the HEC_1_ phase that contains a lower content of the solute atoms should have a closely-matched crystal structure and lattice constant with the solvent TaC lattice due to the reduced atomic position change in the host lattice corresponding to an FCC structure, while the HEC_2_ phase corresponds to the hexagonal structure. The HEC_1_ phase shows a lattice parameter of 0.4499 nm, which is slightly greater than that of TaC (0.4460 nm). Assuming that the HEC_1_ is formed from only transition metal carbides, the lattice parameter should decline as observed in the FCC high-entropy solid solutions in 4-HEC composite (Figure 1a). It is known that the metal-boron bond length is longer than metal-carbon bond length, for example, Ta-C = 2.22 Å [19] and Ta-B = 2.41 Å [20], the expansion of the lattice can be contributed by the addition of B atoms in the formation of an HEC_2_ solid solution. The formation of high-entropy solid solutions HEC_1_ and HEC_2_ that contain all constitutional elements confirms the possibility of processing high-entropy ceramics from a precursor system containing more than one nonmetal atoms and with different crystal structures. The formation of hexagonal structure is likely to be attributed to more B atoms diffusing in the FCC lattice, which induces more severe lattice distortion and consequently leads to the crystal structure change from FCC to a hexagonal structure. In the 5-HEC composite, the FCC structured HEC_1_ solid solution shows a nanohardness of 27.4 GPa and Young’s modulus of 505.8 GPa, which is close to the FCC solid solution phase in 4-HEC (28.4 GPa and 495.2 GPa for nanohardness and Young’s modulus, respectively). 

### 3.2. Effect of Different Starting Particle Sizes

It is well known that the particle size of the precursors is an essential parameter influencing the atomic diffusion and phase evolution during solid-state sintering in PCP [15]. Fine particles promote the solid-state atomic diffusion by reducing the diffusion distance and promote the kinetics of phase transformation [21,22]. To investigate the effect of particle size on the formation of high-entropy ceramics, the same carbide systems with different particle sizes are sintered in PCP (as listed in Table 2). 5-HEC that utilized relative larger particle sizes of solute metal carbides (HfC, Mo_2_C, and TiC) is discussed in the previous section and is denoted as HEC(++) in the following discussion. HEC(fine) and HEC(+) contains precursor carbides with the finest particle sizes and a larger particle size of the solvent carbide (TaC), respectively. 

**Figure 3:**
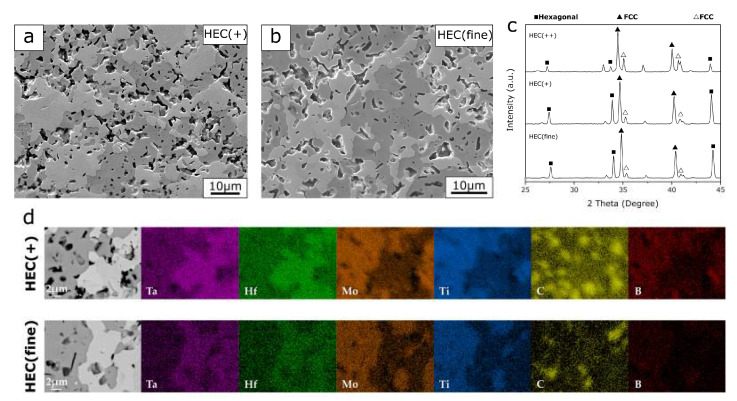
Microstructure (**a**,**b**) and X-ray diffraction phase identification (**c**) and EDS mapping analysis (**d**) of PCP HEC(+) and HEC(fine).

The microstructure of the PCP HEC(+) and HEC(fine) show the presence of two phases in Figure 3. According to the compositional mapping analysis in Figure 3d, all four metal elements are distributed in bright and dark phases. The spot-shaped mapping for C is attributed to porosity in the samples, which possibly caused the diamond polishing agents being introduced during the sample preparation procedure. For both PCP HEC(+) and HEC(fine) sample, the bright phase is rich in Ta and Hf while the dark phase contains a greater amount of Mo and Ti. The quantitative analysis results of the selected areas in Table 3 show that these two phases are high-entropy solid solutions with different elemental compositions. The average atomic ratios are normalized to Ta. The bright phase is rich in Ta, while the dark phase has a higher content of other metals (Ti > Mo > Hf ≈ Ta). Based on the discussion about BED microstructure of different transition metal carbides, the dark region with a higher solute metal content refers to more equilibrium composition and a higher extent of phase transformation towards the high-entropy solid solution. 

The XRD patterns of HEC(+) and HEC(fine) show high similarity (Figure 3c). Similar to the diffractogram of HEC(++), the PCP HEC(+) and HEC(fine) composite reveal diffraction patterns from two FCC and one hexagonal structure. Since the elemental mapping from each region with a constant contrast shows a homogenous distribution of constituent elements, a possible reason why the third phase is not distinguishable in the microstructure is that the two FCC phases have indistinguishable contrast in BED images, which suggests that these phases have a similar atomic composition. As the bright phase with less content of foreign atoms (except Ta), it refers to the component with less lattice distortion, therefore it is extrapolated to retain a FCC crystal structure, whilst the dark phase exhibits a hexagonal structure. 

For the solid-state phase transformations, the starting particle size has been reported to have a strong influence on the reaction kinetics by tuning the contact area between the solid particles [23,24,25]. Therefore, it was expected that PCP HEC(fine) with finest starting particle size should have a more promoted phase transformation than HEC(+) and HEC(++) which were fabricated with the same sintering route and sintering conditions. Comparing HEC(fine) with HEC(+), the results show high similarity in the microstructures and phase composition (Figure 3). Both composites consist of a FCC and a hexagonal crystal structure, with high-entropy solid solutions. With HfC having the lowest formation enthalpy (−1.826 eV) [14] and highest BDE (9.18 g/cm^3^) among the precursors [18], the promotion of the solid-state diffusion can be observed by the increased Hf content in the FCC phase in HEC(fine) than HEC(+) (Hf/Ta = 1 and 0.4, respectively) according to the EDS quantitative results in Table 3. On the other hand, HEC(++) has a complicated phase composition compared to HEC(fine). The Ta-rich phase, representing the least diffusion, was observed in HEC(++) (Figure 1e). A lower fraction of the hexagonal structured high-entropy solid solution in HEC(++) composite can be concluded from the microstructure and lower intensity of X-ray diffraction peaks (for example at 2*θ* = 44.2°) in Figure 3c. Therefore, the degree of diffusion is assumed to be in the order of HEC(++) < HEC(+) ≈ HEC(fine). The results suggest that the particle size of solvent carbide TaC is not as essential as the solute carbides in terms of tailoring the phase composition in the multicomponent carbide system. Due to the porosity and small grain size, the nanoindentation testing of HEC(+) and HEC(fine) composites were obtained from 30 indentations, therefore the data represents the overall mechanical properties of the bulk materials instead of each individual high-entropy phase. Although the PCP HEC(fine) and HEC(+) show the same phase composition and similar microstructure, the HEC(fine) composite sintered from precursors with finest grain size shows improved nanoindentation hardness of 23.1 GPa compared to the PCP HEC(+) composite (21.4 GPa). The experimental hardness value is close to the theoretical hardness calculated from the rule of mixture (23.2 GPa). The enhancement of the hardness caused by utilizing small starting particle size during sintering has been reported before [26]. Moreover, Young’s modulus of HEC(fine) improves from 380.7 GPa for HEC(+) to 401 GPa, suggesting stronger atomic bonding in the HEC(fine) composite.

## 4. Conclusions

A high-entropy ceramic (HEC) composite was synthesized from HfC, Mo_2_C, TaC, and TiC by pulsed current processing (PCP). The PCP 4-HEC composite contained a high-entropy phase and a Ta-rich phase. Both constituents showed a face-centered cubic (FCC) structure and corresponded to the different extent of phase transformation towards a high-entropy phase. By introducing B_4_C in the HEC composite, high-entropy solid solutions that contained all principal elements (including B) were formed. The high-entropy phase with less intense interdiffusion remained FCC structure as the solvent carbide, TaC, while the one with more complete phase transformation experienced crystal structure change from FCC to a hexagonal structure. The results showed the feasibility of synthesizing HEC materials from the multi-principal ceramic system with different crystal structures. 

In order to investigate the effect of different particle sizes of solvent and solute components on the phase composition and properties, HEC composites fabricated from precursors with different size of solvent (TaC) and solute carbides (with face-centered cubic (FCC) structure) were PCP consolidated and characterized. The particle size of the solvent carbides was found to be more essential for the interdiffusion process and final phase compositions than that of the solute carbide. HEC composites processed from the fine particle size of solvent carbides HfC, Mo_2_C, and TiC showed a higher content of the hexagonal structured HEC phase. On the other hand, reducing the TaC particle size to nanoscale showed negligible influence on the phase composition, but resulted in enhancement of the microhardness from 21.4 GPa to 23.1 GPa. 

## Figures and Tables

**Table 1:** The quantitative analysis of the metal atom contents, the crystal structure identification of different phases in 4-HEC and 5-HEC.

Compositional Elements	4-HEC		5-HEC/HEC(++)		
	Ta-Rich Phase (Bright)	High-Entropy Phase (Dark)	Ta-Rich Phase (Bright)	HEC_1_ (Gray)	HEC_2_ (Dark)
Hf	-	2.5	-	0.8	1.7
Mo	Minor	2.4	Minor	1.7	3.3
Ti	-	1.8	-	0.6	7.7
Ta	Major	1	Major	1	1
Crystal structure	FCC	FCC	FCC	FCC	Hexagonal

**Table 2:** Precursors with different particle sizes are utilized to study the effect on the phase evolution.

Component	HEC(++)	HEC(+)	HEC(Fine)
B_4_C	1–7 µm	1–7 µm	1–7 µm
HfC	−325 mesh	<1.25 µm	<1.25 µm
Mo_2_C	−325 mesh	2.6 µm	2.6 µm
TiC	5 µm	2 µm	2 µm
TaC	<1 µm	−325 mesh	<1 µm

**Table 3:** The quantitative analysis of the metal atom contents of different phases in HEC(+) and HEC(fine).

Compositional Elements	HEC(+)		HEC(fine)	
	Bright Phase	Dark Phase	Bright Phase	Dark Phase
Hf	0.4	1.2	1	1.1
Mo	0.8	1.9	0.7	2.0
Ti	0.2	2.3	0.8	2.8
Ta	1	1	1	1
Crystal structure	FCC	Hexagonal	FCC	Hexagonal
